# *N*-Nitro-1*H*-pyrrole-2-carboxamide

**DOI:** 10.1107/S1600536811002455

**Published:** 2011-01-22

**Authors:** Long Liu, Chunlin He, Zengxi Li, Chunshan Li, Xiangping Zhang, Suojiang Zhang

**Affiliations:** a Graduate University of the Chinese Academy of Sciences, Beijing 100049, People’s Republic of China; b State Key Laboratory of Multiphase Complex Systems, Institute of Process Engineering, Chinese Academy of Sciences, Beijing 100190, People’s Republic of China

## Abstract

In the title compound, C_5_H_5_N_3_O_3_, the nitro group is twisted with respect to the amide group, with C—N—N—O torsion angles of 29.0 (2) and −153.66 (14)°. In the crystal, mol­ecules are linked through inter­molecular N—H⋯O and C—H⋯O hydrogen bonds, forming supra­molecular chains along the *a* axis. These chains stack in parallel and form distinct layer motifs in the (001) plane.

## Related literature

For applications of pyrrole derivatives as anti­microbials, see: Mohamed *et al.* (2009 ▶). For the structures of similar pyrrole derivatives, see: Zeng *et al.* (2007 ▶, 2010 ▶); Wang *et al.* (2010 ▶); Ferreira *et al.* (2002 ▶). For the synthesis of *N*,*N*′-dinitro­urea (DNU), see: Goede *et al.* (2001 ▶).
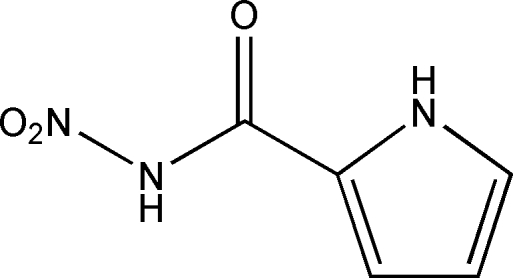


## Experimental

### 

#### Crystal data

C_5_H_5_N_3_O_3_
*M* *_r_* = 155.12
Orthorhombic, 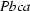

*a* = 9.988 (3) Å
*b* = 6.4547 (17) Å
*c* = 19.184 (6) Å
*V* = 1236.8 (6) Å^3^
*Z* = 8
Mo *K*α radiation
μ = 0.14 mm^−1^
*T* = 133 K
0.47 × 0.43 × 0.20 mm

#### Data collection

Rigaku AFC10/Saturn724+ diffractometer
8849 measured reflections
1402 independent reflections
1214 reflections with *I* > 2σ(*I*)
*R*_int_ = 0.034

#### Refinement

*R*[*F* ^2^ > 2σ(*F* ^2^)] = 0.043
*wR*(*F* ^2^) = 0.106
*S* = 1.00
1402 reflections
108 parameters
H atoms treated by a mixture of independent and constrained refinement
Δρ_max_ = 0.34 e Å^−3^
Δρ_min_ = −0.15 e Å^−3^

### 

Data collection: *CrystalClear* (Rigaku, 2008 ▶); cell refinement: *CrystalClear*; data reduction: *CrystalClear*; program(s) used to solve structure: *SHELXS97* (Sheldrick, 2008 ▶); program(s) used to refine structure: *SHELXL97* (Sheldrick, 2008 ▶); molecular graphics: *X-SEED* (Barbour, 2001 ▶); software used to prepare material for publication: *publCIF* (Westrip, 2010 ▶).

## Supplementary Material

Crystal structure: contains datablocks I, global. DOI: 10.1107/S1600536811002455/fj2382sup1.cif

Structure factors: contains datablocks I. DOI: 10.1107/S1600536811002455/fj2382Isup2.hkl

Additional supplementary materials:  crystallographic information; 3D view; checkCIF report

## Figures and Tables

**Table 1:** Hydrogen-bond geometry (Å, °)

*D*—H⋯*A*	*D*—H	H⋯*A*	*D*⋯*A*	*D*—H⋯*A*
N1—H1*N*⋯O3^i^	0.88 (3)	2.21 (3)	3.001 (2)	150 (2)
N2—H2*N*⋯O1^ii^	0.88 (2)	2.11 (2)	2.982 (2)	171.5 (19)
C3—H3⋯O1^ii^	0.95	2.39	3.269 (2)	154
C3—H3⋯O2^ii^	0.95	2.48	3.245 (2)	138

Symmetry codes: (i) 
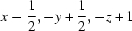
; (ii) 
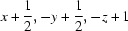
.

**Fig. 1.**
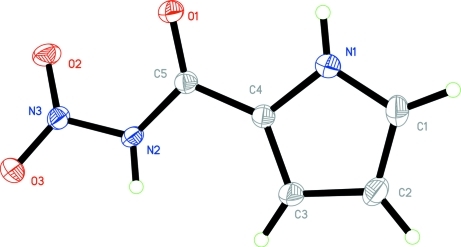
Thermal ellipsoid plot of C5H5N3O3 at the 50% probability level; Hydrogen atoms are drawn as spheres of arbitrary radius.

**Fig. 2.**
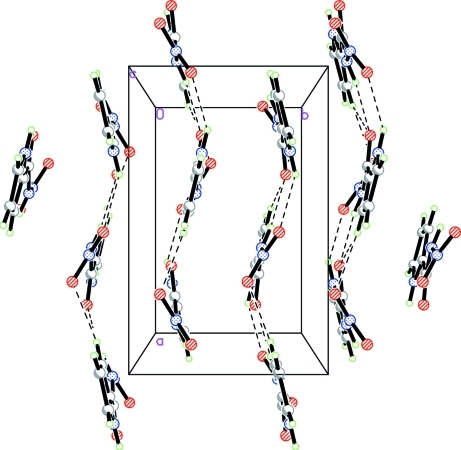
Hydrogen-bonded layer structure.

## supplementary crystallographic information

### Comment

Pyrrole derivatives play an important role in heterocyclic chemistry due to their intrinsic biological activities as antimicrobial agents (Mohamed *et al.*, 2009). The structures of these compounds have been reported extensively, such as 2,3,5-substituted pyrrole derivatives (Ferreira *et al.*, 2002), 1-Benzyl-*N*-methyl-1*H*-pyrrole-2-carboxamide (Zeng *et al.*, 2010), 2-(4,5-dibromo-1*H*-pyrrole-2-carboxamido) propionate (Zeng *et al.*, 2007) and Tetraethyl 1,1'-(ethane-1,2-diyl)bis(2,5- dimethyl-1*H*-pyrrole-3,4-dicarboxylate) (Wang *et al.*, 2010).

The bond length of N2—C5 for the title compound (1.404 (2) Å) is about 0.07 Å longer than compound 1-Benzyl-*N*-methyl-1*H*-pyrrole-2-carboxamide (1.334 (3) Å) (Zeng *et al.*, 2010) (Fig. 1). The unit is nearly co-planar with the twist happens at nitro group (C5—N2—N3—O2 = 29.0 (2), C5—N2—N3—O3 = -153.66 (14)), the maximum deviation of other torsions is C3—C4—C5—N2 = -8.6 (3)°.

In the crystal structure (Fig. 2), molecules are connected through N1—H1N···O3, N2—H2N···O1, C3—H3···O1, C3—H3···O3 (Table 1) hydrogen bonds to form one-dimensional supramolecular chains along the *a* axis. These supramolecular chains stack in parallel and form distinct layer motif in (0 0 1) plane.

### Experimental

Pyrrole (0.67 g, 0.01 mol) was added to a solution of *N*,*N*'-dinitrourea (DNU) (1.5 g, 0.01 mol) dissolved in acetonitrile (10 ml), stirred at room temperature for 24 h, the crude compound was obtained after acetonitrile was evaporated. Then the products were dissoved in ethyl acetate, colourless crystals suitable for X-ray crystal diffraction were obtained by slow evaporation of the solution at room temperature. DNU was synthesized according to the literautre (Goede *et al.*, 2001).

### Refinement

The hydrogen atoms bonded to N1 and N2 were located from a difference Fourier maps and refined isotropically with N—H = 0.88 (3) Å and 0.88 (2) Å respectively. The remaining hydrogen atoms were geometrically positioned (all C—H = 0.9500 Å).

### Crystal data

**Table:**

C_5_H_5_N_3_O_3_	*F*(000) = 640
*M**_r_* = 155.12	*D*_x_ = 1.666 Mg m^−^^3^
Orthorhombic, *P**b**c**a*	Mo *K*α radiation, λ = 0.71073 Å
Hall symbol: -P 2ac 2ab	Cell parameters from 3166 reflections
*a* = 9.988 (3) Å	θ = 3.2–27.5°
*b* = 6.4547 (17) Å	µ = 0.14 mm^−^^1^
*c* = 19.184 (6) Å	*T* = 133 K
*V* = 1236.8 (6) Å^3^	Platelet, colourless
*Z* = 8	0.47 × 0.43 × 0.20 mm

### Data collection

**Table:**

Rigaku AFC10/Saturn724+ diffractometer	1214 reflections with *I* > 2σ(*I*)
Radiation source: Rotating Anode	*R*_int_ = 0.034
graphite	θ_max_ = 27.5°, θ_min_ = 3.9°
Detector resolution: 28.5714 pixels mm^-1^	*h* = −12→12
φ and ω scans	*k* = −8→8
8849 measured reflections	*l* = −24→24
1402 independent reflections	

### Refinement

**Table:**

Refinement on *F*^2^	Primary atom site location: structure-invariant direct methods
Least-squares matrix: full	Secondary atom site location: difference Fourier map
*R*[*F*^2^ > 2σ(*F*^2^)] = 0.043	Hydrogen site location: inferred from neighbouring sites
*wR*(*F*^2^) = 0.106	H atoms treated by a mixture of independent and constrained refinement
*S* = 1.00	*w* = 1/[σ^2^(*F*_o_^2^) + (0.0596*P*)^2^ + 0.536*P*] where *P* = (*F*_o_^2^ + 2*F*_c_^2^)/3
1402 reflections	(Δ/σ)_max_ < 0.001
108 parameters	Δρ_max_ = 0.34 e Å^−^^3^
0 restraints	Δρ_min_ = −0.15 e Å^−^^3^

### Special details

**Table:**

Geometry. All e.s.d.'s (except the e.s.d. in the dihedral angle between two l.s. planes) are estimated using the full covariance matrix. The cell e.s.d.'s are taken into account individually in the estimation of e.s.d.'s in distances, angles and torsion angles; correlations between e.s.d.'s in cell parameters are only used when they are defined by crystal symmetry. An approximate (isotropic) treatment of cell e.s.d.'s is used for estimating e.s.d.'s involving l.s. planes.
Refinement. Refinement of *F*^2^ against ALL reflections. The weighted *R*-factor *wR* and goodness of fit *S* are based on *F*^2^, conventional *R*-factors *R* are based on *F*, with *F* set to zero for negative *F*^2^. The threshold expression of *F*^2^ > σ(*F*^2^) is used only for calculating *R*-factors(gt) *etc*. and is not relevant to the choice of reflections for refinement. *R*-factors based on *F*^2^ are statistically about twice as large as those based on *F*, and *R*- factors based on ALL data will be even larger.

### Fractional atomic coordinates and isotropic or equivalent isotropic displacement parameters (Å^2^)

**Table:**

	*x*	*y*	*z*	*U*_iso_*/*U*_eq_	
O1	0.17512 (12)	0.34028 (18)	0.50500 (5)	0.0219 (3)	
O2	0.27334 (11)	0.3909 (2)	0.37665 (6)	0.0256 (3)	
O3	0.45611 (12)	0.21230 (19)	0.36431 (6)	0.0255 (3)	
N1	0.25340 (14)	0.3489 (2)	0.64493 (7)	0.0207 (3)	
N2	0.39562 (13)	0.3045 (2)	0.47037 (7)	0.0182 (3)	
N3	0.37143 (13)	0.3012 (2)	0.39910 (7)	0.0178 (3)	
C1	0.31849 (18)	0.3310 (3)	0.70652 (8)	0.0235 (4)	
H1	0.2800	0.3496	0.7514	0.028*	
C2	0.44956 (17)	0.2815 (3)	0.69312 (8)	0.0240 (4)	
H2	0.5173	0.2581	0.7270	0.029*	
C3	0.46594 (16)	0.2715 (2)	0.62056 (8)	0.0198 (4)	
H3	0.5464	0.2417	0.5962	0.024*	
C4	0.34176 (15)	0.3137 (2)	0.59132 (8)	0.0160 (3)	
C5	0.29335 (15)	0.3204 (2)	0.52027 (8)	0.0158 (3)	
H1N	0.169 (3)	0.380 (4)	0.6375 (12)	0.049 (7)*	
H2N	0.474 (2)	0.250 (3)	0.4794 (11)	0.032 (6)*	

### Atomic displacement parameters (Å^2^)

**Table:**

	*U*^11^	*U*^22^	*U*^33^	*U*^12^	*U*^13^	*U*^23^
O1	0.0140 (6)	0.0339 (7)	0.0177 (5)	−0.0002 (5)	−0.0004 (4)	0.0003 (5)
O2	0.0181 (6)	0.0398 (7)	0.0188 (6)	0.0049 (5)	−0.0022 (4)	0.0071 (5)
O3	0.0198 (6)	0.0381 (7)	0.0187 (6)	0.0050 (5)	0.0027 (5)	−0.0052 (5)
N1	0.0164 (7)	0.0276 (7)	0.0180 (6)	0.0021 (6)	0.0007 (5)	0.0002 (6)
N2	0.0132 (6)	0.0281 (7)	0.0133 (6)	0.0016 (5)	−0.0022 (5)	0.0011 (5)
N3	0.0153 (6)	0.0241 (7)	0.0141 (6)	−0.0022 (5)	0.0004 (5)	0.0013 (5)
C1	0.0273 (9)	0.0286 (9)	0.0145 (7)	−0.0002 (7)	0.0011 (6)	0.0000 (6)
C2	0.0223 (8)	0.0315 (9)	0.0183 (8)	−0.0013 (7)	−0.0053 (6)	0.0015 (6)
C3	0.0146 (7)	0.0257 (8)	0.0191 (8)	−0.0008 (6)	−0.0001 (6)	0.0010 (6)
C4	0.0147 (7)	0.0176 (7)	0.0158 (7)	−0.0013 (6)	0.0003 (6)	−0.0001 (5)
C5	0.0148 (7)	0.0164 (7)	0.0163 (7)	−0.0011 (6)	−0.0002 (5)	−0.0002 (6)

### Geometric parameters (Å, °)

**Table:**

O1—C5	1.2234 (19)	N2—H2N	0.88 (2)
O2—N3	1.2167 (17)	C1—C2	1.372 (2)
O3—N3	1.2206 (17)	C1—H1	0.9500
N1—C1	1.354 (2)	C2—C3	1.403 (2)
N1—C4	1.374 (2)	C2—H2	0.9500
N1—H1N	0.88 (3)	C3—C4	1.388 (2)
N2—N3	1.3886 (18)	C3—H3	0.9500
N2—C5	1.404 (2)	C4—C5	1.447 (2)
			
C1—N1—C4	109.32 (14)	C1—C2—C3	107.93 (15)
C1—N1—H1N	128.4 (16)	C1—C2—H2	126.0
C4—N1—H1N	122.3 (16)	C3—C2—H2	126.0
N3—N2—C5	123.09 (13)	C4—C3—C2	106.72 (14)
N3—N2—H2N	110.2 (14)	C4—C3—H3	126.6
C5—N2—H2N	123.1 (14)	C2—C3—H3	126.6
O2—N3—O3	126.02 (14)	N1—C4—C3	107.68 (13)
O2—N3—N2	118.77 (13)	N1—C4—C5	119.04 (14)
O3—N3—N2	115.15 (13)	C3—C4—C5	133.25 (14)
N1—C1—C2	108.33 (14)	O1—C5—N2	123.14 (14)
N1—C1—H1	125.8	O1—C5—C4	123.46 (14)
C2—C1—H1	125.8	N2—C5—C4	113.39 (13)
			
C5—N2—N3—O2	29.0 (2)	C2—C3—C4—C5	−177.88 (16)
C5—N2—N3—O3	−153.66 (14)	N3—N2—C5—O1	−2.7 (2)
C4—N1—C1—C2	−0.71 (19)	N3—N2—C5—C4	177.98 (13)
N1—C1—C2—C3	0.9 (2)	N1—C4—C5—O1	−5.8 (2)
C1—C2—C3—C4	−0.67 (19)	C3—C4—C5—O1	172.13 (17)
C1—N1—C4—C3	0.28 (18)	N1—C4—C5—N2	173.46 (13)
C1—N1—C4—C5	178.72 (14)	C3—C4—C5—N2	−8.6 (3)
C2—C3—C4—N1	0.24 (18)		

### Hydrogen-bond geometry (Å, °)

**Table:**

*D*—H···*A*	*D*—H	H···*A*	*D*···*A*	*D*—H···*A*
N1—H1N···O3^i^	0.88 (3)	2.21 (3)	3.001 (2)	150 (2)
N2—H2N···O1^ii^	0.88 (2)	2.11 (2)	2.982 (2)	171.5 (19)
C3—H3···O1^ii^	0.95	2.39	3.269 (2)	154
C3—H3···O2^ii^	0.95	2.48	3.245 (2)	138

Symmetry codes: (i) *x*−1/2, −*y*+1/2, −*z*+1; (ii) *x*+1/2, −*y*+1/2, −*z*+1.
